# Socioeconomic factors contributing to exclusion of women from maternal health benefit in Abuja, Nigeria

**DOI:** 10.4102/curationis.v38i1.1272

**Published:** 2015-07-21

**Authors:** Tajudeen O. Oyewale, Thandisizwe R. Mavundla

**Affiliations:** 1 Department of Health Studies, University of South Africa, South Africa

## Abstract

**Background:**

An understanding of the predictive effect of socioeconomic characteristics (SECs) of women on maternal healthcare service utilisation is essential in order to maximise maternal health benefits and outcomes for the newborn.

**Objectives:**

To describe how SECs of women contribute to their exclusion from maternal health benefits in Abuja Municipal Areas Council (AMAC) in Abuja, Nigeria.

**Method:**

A non-experimental, facility-based cross-sectional survey was done. Data were collected from 384 respondents using a structured interviewer-administered questionnaire. The participants were sampled randomly at antenatal care (ANC) clinics in the five district hospitals in AMAC. Data analysis included descriptive statistics, cross-tabulations and measures of inequality. Logistic regression analysis was used to test the relationship between SECs (predictors) and maternal healthcare service utilisation.

**Results:**

There were differentials in the utilisation of maternal healthcare services (ANC, delivery care, post natal care [PNC] and contraceptive services) amongst women with different SECs; and the payment system for maternal healthcare services was regressive. There were inconsistencies in the predictive effect of the SECs of women included in this study (age, education, birth order, location of residence, income group and coverage by health insurance) on maternal healthcare service utilisation when considered independently (bivariate analysis) as opposed to when considered together (logistic regression), with the exception of birth order, which showed consistent effect.

**Conclusion:**

SECs of women were predictive factors of utilisation of maternal healthcare services. There is a need for targeted policy measures and programme actions toward multiple SECs of women in their natural co-existing state in order to optimise maternal health benefits.

## Introduction

Equitable access to and utilisation of maternal healthcare services are critical inputs toward the achievement of Millennium Development Goal (MDG) 5 in Nigeria. Currently, the maternal mortality ratio (MMR) in Nigeria is estimated to be 545 deaths per 100 000 live births (National Planning Commission [NPC] & ICF Macro 2009:237) and further analysis of this information indicated that there are equity concerns related to the socioeconomic characteristics (SECs) of women. These concerns informed the equity focus of Nigeria's revised national health policy, of which the underlying principles and values included: (1) social justice and equity, and the ideals of freedom and opportunity; (2) access to quality and affordable healthcare as a human right; and (3) equity in healthcare and in health for all Nigerians (Federal Ministry of Health [FMOH] 2004:4).

Proper healthcare during pregnancy, delivery and the postnatal period is important for health outcomes for mothers and the newborn. It is worrisome however, that 61% of pregnant women in Nigeria received no antenatal care (ANC) and only 38% of women had skilled delivery care (NPC & MEASURE DHS 2013:22). Earlier studies had reported that 56% of mothers did not receive postnatal care (PNC) (NPC & ICF Macro 2009:135) and less than one in five sexually-active Nigerian women used modern contraceptive methods (FMOH 2008:132; National Bureau of Statistics [NBS], UNICEF & UNFPA 2013:130; NPC & ICF Macro 2009:69; NPC & MEASURE DHS 2013:16)

**FIGURE 1:**
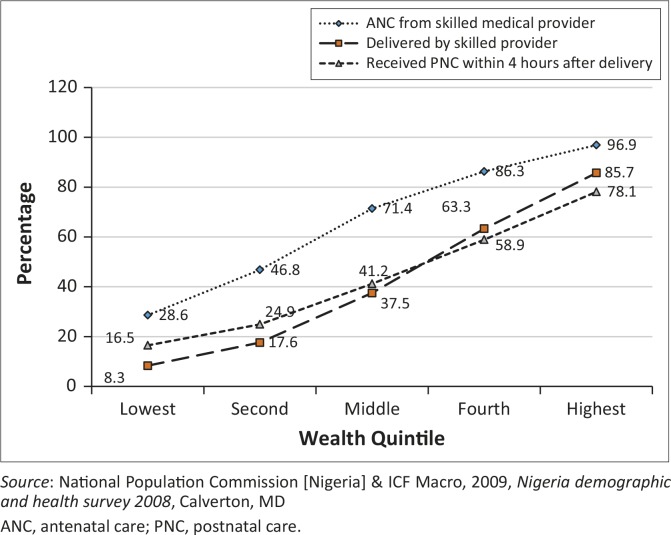
Pattern of exclusion of pregnant women from maternal healthcare services in Nigeria.

The uptake of ANC, delivery care and PNC services varied amongst women of different SECs in Nigeria (NBS *et al*. 2013:130–145; NPC & ICF Macro 2009:126–136; NPC & MEASURE DHS 2013:15–22). The construction of access to maternal healthcare services amongst women of different wealth quintiles in Nigeria (Figure 1) depicts a massive deprivation pattern of exclusion from healthcare services, with a large proportion of the population being deprived of care (World Health Organization [WHO] 2005:29).

The impact of the different SECs of women on the different maternal healthcare services varied. A sub-national study in Emevor, Delta State Nigeria (Awusi, Anyanwu & Okeke 2009:22) reported that maternal age was associated significantly (*p* < 0.05) with utilisation of ANC; and that 87% of women aged less than 30 utilised ANC compared with 13% of those older than 30. Doctor and Dahiru (2010:42) had reported a statistically-significant relationship (*p* < 0.001) between age and delivery by non-skilled birth attendants (NSBA) in Northern Nigeria. Another study (Oguntunde *et al*. 2010:91) in Northern Nigeria, however, reported that age was not associated statistically with utilisation of ANC. Women in Nigeria (Awusi *et al*. 2009:23) and Ethiopia (Mekonnen & Mekonnen 2003:375–376), with limited or no past experience related to pregnancy and child birth, were reported to be more likely to seek ANC and professional assistance at delivery. A study in Kaduna, Nigeria (Butawa *et al*. 2010:75), argued that the number of schooling years completed by women influenced their perception and utilisation of healthcare services. In Ghana, Adanu (2010:155) reported that women with the highest education status had a significant chance (*p* < 0.001) of being attended by doctors at an ANC. A similar effect was reported for the use of contraceptive services in Nigeria (Avidime *et al*. 2010:69). However, formal schooling was not significant (*p* = 0.106) with regard to the receipt of NSBA in northern Nigeria (Doctor & Dahiru 2010:42). Earlier studies (Lynch *et al*. 2000:1201; Hernández-Quevedo, Masseria & Mossialos 2000:9) indicated that household income influences health outcomes. In Wirth *et al*. (2006:523), the ‘non-poor’ (defined by household income) in Kenya were twice as likely as the poor to have a skilled attendant at their birth.

The poor transportation system and low public expenditure on health worsen the exclusion of women from maternal healthcare services in Nigeria. According to the Health Reform Foundation of Nigeria [HERFON] 2006:201), the general expenditure on health by the Nigerian government over the period of 1998 to 2002 was 20% of the Total Health Expenditure. In 2003, public health expenditure in Nigeria was less than $8 per capita compared with the $34 recommended globally for low-income countries (FMOH 2004:2). Private health expenditure accounted for 69% of the Total Health Expenditure in Nigeria (HERFON 2006:201). It is therefore not surprising that maternal mortality in Nigeria remains a concern (WHO 2010:17).

### Conceptual framework

The framework for analysing the determinants of maternal mortality in McCarthy (1997:S17) and the behavioural model of health services utilisation (Andersen & Newman 2005:12) were used as the conceptual framework for this study. In the exploration of how SECs of women influences the proximate determinant of maternal mortality, McCarthy (1997:S18) argued that any intervention to reduce maternal death (the ultimate maternal health benefit) must pass through one of three pathways to: (1) reduce the number of pregnancies; (2) reduce the number of complications; or (3) reduce the likelihood that a complication will result in death. The three pathways were contextualised into the four maternal healthcare services in this study (Table 1).

**TABLE 1:** Contextual maternal healthcare services to reduce maternal deaths.

Pathway to reduce maternal death	Contextual maternal healthcare services in this study
Reduce number of pregnancies.	Contraceptive services.
Reduce number of complications.	Antenatal care (ANC).
Reduce likelihood that a complication will result in death.	Skilled delivery care at birth and Postnatal care (PNC).

*Source*: McCarthy, J., 1997, ‘The conceptual framework of the PMM network’, *International Journal of Gynecology and Obstetrics* 59(Suppl 2), S15–S21

The behavioural model of health services utilisation (Andersen & Newman 2005:12) guided further insight into how SECs of women contribute to exclusion from maternal health benefits. The model was based on the assumption that utilisation of (maternal) health services is dependent on three categories of individual determinants, namely: (1) the predisposition of the individual to use the service; (2) the ability of the individual to secure the service (enabling factor); and (3) the illness level of the individual. This study focused on the predisposing and enabling factors, as the physiological state of pregnancy already defined the ‘illness level’ amongst the study population.

### Problem statement

Whilst several efforts and investments were made to improve maternal health in Nigeria, little is known about the complex interplay of the SECs of women on maternal healthcare service utilisation. Available evidence was limited to the consequence of economic hardship on health-seeking behaviours (Akpomuvie 2010:48). A study to describe the impact of women's SECs on the determinants of maternal mortality is therefore required in order to understand the contribution of SECs of women to maternal health benefits in AMAC, Abuja, Nigeria. This study will contribute toward the achievement of MDG 5 and national targets on maternal health in Nigeria.

## Research design

The research sought to describe how SECs of women contribute to their exclusion from maternal health benefits in AMAC, Abuja, Nigeria. The objectives of the research are to describe the utilisation of maternal healthcare services amongst women with different SECs; describe the pattern of inequality in the utilisation of maternal healthcare services amongst women of different SECs; and describe the predictive effects of SECs of women on the utilisation of maternal healthcare services.

### Significance

The maternal health benefit encompasses interventions to reduce death related to pregnancy and its complications. Understanding the predictors (socioeconomic characteristics) of utilisation of these interventions is important with regard to optimisation of the outcome for women. The researchers are of the opinion that policy and programme review informed by the effect of multiple predictors will result in a more effective and equitable outcome than those reviews that consider the influence of the predictors independently. This means that maternal health benefit is not only dependent on availability of maternal healthcare services, but also on the predisposing and enabling factors of utilisation amongst women.

### Research approach and method

#### Design

A non-experimental, facility-based cross-sectional survey (Araoye 2003:55; Babbie & Mouton 2004:180; Grimes & Schulz 2002:145) was done amongst pregnant women in AMAC. Inclusion criteria were pregnant women with a past pregnancy history, irrespective of past pregnancy outcome, who were currently registered in the study facilities. Exclusion criteria were pregnant women who were newly enrolled in the study facilities but had yet to be included in the ANC register.

#### Sampling and criteria for sample selection

The five (100%) district hospitals in AMAC were included in the study. Using the formula for sample size estimation (Araoye 2003:119), the calculated sample size was 384, but was increased to 385 to allow for equal allocation per facility and adequate representation of all districts in AMAC. In each hospital, proportionate allocation of samples was done per clinic day. Respondents were selected through simple ballot at the ANC clinic by picking ‘Yes’ or ‘No’ ballots. Where a selected woman declined, another eligible woman identified by simple ballot was interviewed until the allocation for the clinic day was met.

#### Data collection

An interviewer-administered questionnaire informed by the conceptual framework of this study was used for data collection as 46% of Nigerian women were not literate (NPC & ICF Macro 2009:35). The questionnaire was composed mostly of closed-ended question items with categorical answer options that had been used in an earlier study on maternal healthcare in Nigeria (NPC & ICF Macro 2009:527−599). Informed by the pilot of the questionnaire amongst 53 pregnant women at the district hospital in Bwari area council, adjacent to AMAC, open-ended questions for household income and health expenditure were included in the questionnaire. Data were collected by five well-trained professional personnel who were familiar with the local context and the subject matter. Prior to data collection, the research personnel were oriented to the purpose of the research, the findings from the pilot exercise and the content of the questionnaire. Respondents were interviewed over a period of one week in each facility so as to avoid double counting.

#### Data analysis

All 385 questionnaires were returned, but one questionnaire was excluded from analysis as the majority of the questions were not answered. Data analysis was done with SPSS version 20 (IBM Corp., Armonk, NY 2011) and included descriptive statistics, cross-tabulations, measures of inequality and logistic regression. The descriptive statistics included mean, median, standard deviation (SD), range and percentages (Araoye 2003:168; Kumaranayake *et al*. 2008: 10–16). A three-scale category was established for annual household income based on the range (Araoye 2003:177) and knowledge of contraceptive methods based on the mean and SD (Kirkwood & Sterne 2003:45–47). Six SECs of respondents (Wirth *et al*. 2006:520) and specific utilisation measures for the four maternal healthcare services as defined in NPC and ICF Macro (2009:66–134) were included in the bivariate analysis (Table 2). Cross-tabulation of the dependent and independent variables were done, with differences being interpreted where *p* < 0.05.

**TABLE 2:** Classification of variables in bivariate and multi­variate analysis and their respective values.

Variable	Value
**Outcome variables (Measure of utilisation of maternal healthcare services)**	
Contraceptive services	0 = Used modern contraceptive methods
	1 = Did not use modern contraceptive methods
Antenatal care (ANC) service	0 = Received ANC from skilled medical providers
	1 = Did not receive ANC from skilled medical providers
Delivery care	0 = Had skilled attendants at birth
	1 = Did not have skilled attendants at birth
Postnatal care (PNC) services	0 = Receive PNC from medical personnel at any time after delivery
	1 = Did not receive PNC from medical personnel
**Exposure variables (Socioeconomic stratifiers)**	
Age	1 = Adolescent (younger than 20 years)
	2 = Young adult (20–34 years)
	3 = Adult (35 years and older)
Education	0 = None / Primary education (also if primary education was not completed)
	1 = Secondary education (also if secondary education was not completed)
	2 = Post-secondary education
Birth order	1 = Once
	2 = More than once
Residence	0 = Rural
	1 = Urban
Income group	1 = Lower
	2 = Average
	3 = Higher
Insurance status	0 = Not covered (No)
	1 = Covered (Yes)

The measure of inequality in the utilisation of maternal healthcare services was the concentration index which lies between -1 and +1 and defined in relation to the concentration curve (O’ Donnell *et al*. 2008:95; Onwujekwe *et al*. 2008:4; Wagstaff, Paci & Van Doorslaer 1991:548). Vertical equity in direct payments for healthcare amongst respondents was determined using the Kakwani index (*K*) defined in relation to the payment concentration curve and the Lorenz curve (De Maio 2007:851; Mastilica & Bož ikov 1999:154; Reinhardt 2010:2). If payments were levied strictly in proportion to income, the payment concentration curve and the Lorenz curve would coincide and *K* = 0. If payment systems were regressive, *K* < 1; otherwise progressive if *K* > 1.

Logistic regression coefficient (β) and odds ratio (OR) were used in the determination of the association between the exposure and outcome variables (Burns & Burns 2008:573; Kirkwood & Sterne 2003:197) in the logistic regression. The classification of the variables and their respective values are defined in Table 2. The OR represented the change in the odds of being in one category of the outcome variable over the reference category and interpreted where *p* values were <0.05 (Burns & Burns 2008:579).

The equation for the logistic regression analysis (Kirkwood & Sterne 2003:197) in this study was the following:

Log odds of outcome=β0+β1x1+β2x2+…+βpxp [Eqn 1]

## Ethical considerations

Ethical approval for this research was secured from the University of South Africa's (UNISA) Health Studies Research Ethics Committee (project # 3531-159-2) and the Federal Capital Territory (FCT) Abuja Health Research Ethics Committee (protocol approval # FHREC/2012/01/03/21-3-12) in Nigeria. In addition, ethical considerations (Mouton 2001:243–245; Patten 2002:25) relating to the right to full disclosure and informed consent were maintained. Participants had the right to refuse to participate in the research, including the right to withdraw after securing consent. Other ethical considerations included the right to anonymity by keeping the identity of the respondents secret; the right to confidentiality by not linking collected information to respondents; and the right not to be harmed in any manner.

## Trustworthiness

### Reliability

The reliability of the measurement was assured through investigator consistency by using trained professional data collection personnel. This was reinforced by standardising the conditions for measurements by interviewing the women at ANC clinics so as to limit external influence (Cooper & Schindler 2003:239). In addition, the survey instrument was finalised based on the findings from the pilot and the research subjects (pregnant women) were relevant to the study (Babbie & Mouton 2004:121).

### Validity

The face validity of the research instrument lay in the fact that the question items were derived from an existing questionnaire with established face and content validity (Araoye 2003:151; Patten 2002:55). Content validity of the survey instrument was further assured by basing the questionnaire design on the conceptual framework for the study (Araoye 2003:153; Babbie & Mouton 2004:123). The probability sampling technique in subject selection and the calculation of large sample size for this study contributed to external validity (Araoye 2003:151; Cooper & Schindler 2003:231). Other measures to assure external validity included the design of the questionnaire based on rules for questionnaire construction, the pre-testing of the instrument and ethical rigour, especially with regard to assuring anonymity (the questionnaire identification number cannot be traced to particular respondents).

## Discussion of results

### Socioeconomic characteristics

The socio-demographic characteristics of the respondents are presented in Table 3. The median annual household income was Naira 699 996.00. Regarding income category, 285 (74%) respondents were categorised into the lower income group, 44 (12%) into the average income group and 55 (14%) into the higher income group. Amongst the 91 (24%) respondents who were covered by health insurance, 47 (52%) had public health insurance, 28 (31%) had private or commercial health insurance, 4 (4%) had community-based health insurance and 12 (13%) had other forms of insurance.

**TABLE 3:** Socio-demographic characteristics of the respondents (*N* = 384).

Characteristics	Distribution	Percentage
**Age**		
Adolescent (younger than 20 years)	16	4.2
Young adult (20–34 years)	295	76.8
Adult (35 years and older)	73	19
**Highest educational level**		
None / Primary Education	25	6.5
Secondary	107	27.9
Post-secondary education	252	65.6
**Marital status**		
Single	35	9.1
Married	333	86.7
Divorced	3	0.8
Widow	13	3.4
**Number of children of respondents**		
None	87	22.7
1–2 children	182	47.4
3–4 children	102	26.6
5 children or more	13	3.4
**Location where respondent lived during the last pregnancy **		
Urban	331	86.2
Rural	53	13.8
**Ethnic group of respondents**		
Hausa	122	31.8
Ibo	111	28.9
Yoruba	93	24.2
Others	58	15.1
**Number of births by respondents, including last pregnancy (birth order)**		
1 birth	153	39.8
2 or more births	231	60.2

### Description of maternal healthcare services utilisation

The utilisation of maternal healthcare services varied amongst women of different SECs in AMAC (Table 4). Overall, 295 (77%) women utilised ANC, 249 (65%) received skilled delivery care and 245 (64%) received PNC. Although 307 (80%) respondents were aware of contraceptives, the mean knowledge score of contraceptive methods was 2.24 (SD ± 1.88) out of a total score of 13. Amongst the respondents, 52 (14%) had good knowledge of contraceptive methods, 230 (60%) had average knowledge and 102 (27%) had poor knowledge. It was therefore consistent that only 199 (52%) respondents had used modern contraceptive methods.

**TABLE 4:** Cross-tabulation of maternal healthcare service utilisation against selected socioeconomic characteristics (SEC) of respondents.

Maternal Healthcare Service	Age (%)			Birth Order (%)		Education Level (Percentage)			Location of Residence (Percentage)		Insurance Coverage (%)		Income Group (%)		
	< 20 years	20–34 years	35 years and older	1 Birth	>1 Birth	None / Primary	Secondary	Post-Secondary	Urban	Rural	Covered	Not Covered	Lower	Average	Higher
**Contraceptive services**															
Used modern contraceptive methods (*n* = 199)	1.0	81.9	17.1	38.2	61.8	2	16.6	81.4	94.5	5.5	27.6	72.4	68.3	13.1	18.6
Did not use modern contraceptive methods (*n*= 185)	7.6	71.4	21.1	41.6	58.4	11.4	40	48.6	77.3	22.7	19.5	80.5	80.5	9.7	9.7
**Chi-square**	**12.106**			**0.471**		**47.394**			**23.771**		**3.547**		**8.112**		
***p*-value**	**0.002**			**0.493**		**0.000**			**0.000**		**0.060**		**0.017**		
**Antenatal care (ANC) service**															
Received ANC from medical skilled providers (*n*= 295)	0.7	79.3	20	28.5	71.5	5.1	26.8	68.1	86.1	13.9	26.4	73.6	73.9	10.5	15.6
Did not receive ANC from medical skilled providers (*n*= 89)	15.7	68.6	15.7	77.5	22.5	11.2	31.5	57.3	86.5	13.5	14.6	85.4	75.3	14.6	10.1
**Chi-square**	**38.870**			**68.640**		**5.734**			**0.010**		**5.295**		**2.454**		
***p*-value**	**0.000**			**0.000**		**0.057**			**0.921**		**0.021**		**0.294**		
**Delivery care services**															
Had skilled attendants at birth (*n* = 249)	0.8	80.3	18.9	31.7	68.3	4.4	25.7	69.9	88.0	12.0	29.7	70.3	71.9	10.8	17.3
Did not have skilled attendants at birth (*n* = 135)	10.4	70.4	19.3	54.8	45.2	10.4	31.9	57.8	83.0	17.0	12.6	87.4	78.5	12.6	8.9
**Chi-square**	**20.365**			**19.468**		**7.906**			**1.831**		**14.200**		**5.045**		
***p*-value**	**0.000**			**0.000**		**0.019**			**0.176**		**0.000**		**0.080**		
**Postnatal care (PNC)**															
Received PNC from medical personnel (*n* = 245)	0.8	81.2	18	32.2	67.8	4.1	25.3	70.6	87.8	12.2	29.4	70.6	72.2	10.6	17.1
Did not receive PNC from medical personnel (*n*=139)	10.1	69.1	20.9	53.2	46.8	10.8	32.4	56.8	83.5	16.5	13.7	86.3	77.7	12.9	9.4
**Chi-square**	**20.334**			**16.306**		**10.288**			**1.38**		**12.118**		**4.536**		
***p*-value**	**0.000**			**0.000**		**0.006**			**0.240**		**0.000**		**0.104**		

### Maternal age

The differential in the utilisation of modern contraceptive methods (*p* = 0.002), ANC (*p* = 0.000), skilled delivery care (*p* = 0.000) and PNC (*p* = 0.000) amongst women of different age groups was statistically significant. Similar to earlier studies in Kenya (Okech, Wawire & Mburu 2011:29) and Nigeria (NBS *et al*. 2013:129; NPC & MEASURE DHS 2013:15), the majority of adolescent respondents in AMAC were excluded from the utilisation of the four maternal healthcare services. This situation reinforces the earlier argument by the WHO (2011:12), which stated that socioeconomic inequities resulting from limited access to funds, requirement of parental consent, societal exclusion because of early marriage or sexual coercion, or discrimination by judgmental health workers faced by adolescents, limits their access to maternal healthcare.

### Birth order

Unlike the trend in earlier reports (Awusi *et al*. 2009:23; Mekonnen & Mekonnen 2003:375–376), the majority of respondents who utilised the four maternal healthcare services in AMAC had experienced more than one birth. The observed trend was, however, similar to the use of modern contraceptive methods in Kenya (Okech *et al*. 2011:30). Other than the use of modern contraceptive methods (*p* = 0.493), the differential in the use of ANC (*p* = 0.000), skilled delivery care (*p* = 0.000) and PNC (*p* = 0.000) amongst respondents with different birth order was statistically significant.

### Maternal education

As expected (NBS *et al*. 2013:130–145; NPC & ICF Macro 2009:69–136; NPC & MEASURE DHS 2013:16–22), the proportion of respondents who used the four maternal healthcare services in this study increased along with an increasing level of maternal education. Likewise in Kenya, Okech *et al*. (2011:30) reported an increasing proportion of utilisation of modern contraceptive methods amongst women with an increasing level of education. The use of modern contraceptive methods (*p* = 0.000), skilled delivery care (*p* = 0.019) and PNC (*p* = 0.006) amongst respondents with different educational levels was significant. The use of ANC (*p* = 0.057) amongst women with different education levels was, however, not statistically significant.

### Location of residence

Consistent with earlier studies in Nigeria (NBS *et al*. 2013:129–148; NPC & MEASURE DHS 2013:15–22) and Ethiopia (Mekonnen & Mekonnen 2003:376–378), the majority of respondents who used the four maternal healthcare services lived in urban settings. However, only the differential in the use of modern contraceptive methods (*p* = 0.000) amongst women resident in different locations (urban/rural) in AMAC was significant. The use of ANC (*p* = 0.921), skilled delivery care (*p* = 0.176) and PNC (*p* = 0.240) were not influenced significantly by location of residence. Divergent influences of location of residence on the use of the different maternal healthcare services were reported in Jamaica and India. According to Say and Raine (2007:815), urban women in Jamaica were significantly less likely (*p* < 0.05) to attend ANC than rural women. In contrast, studies from India, reviewed in Say and Raine (2007:814), either reported no difference or a significant difference (*p* < 0.01) in the utilisation of skilled health workers at delivery in favour of urban women.

### Health insurance coverage

In general, membership of a health insurance plan is expected to contribute toward higher service utilisation amongst scheme members (Soors, Waelkens & Criel 2008:160). In this study, however, overall coverage of health insurance was low and a lower proportion of respondents who utilised the four maternal healthcare services were covered by health insurance. These findings may be attributed to the documented equity concerns with regard to the coverage and benefit of health insurance in low- and middle-income countries (Meng *et al*. 2011:94). Other than the use of modern contraceptive methods (*p* = 0.060), procured largely outside of health facilities, the differential in the use of ANC (*p* = 0.021), skilled delivery care (*p* = 0.000) and PNC (*p* = 0.000) amongst respondents with different health insurance coverage were statistically significant. Amongst the 201 (52%) respondents who had procured contraceptives in this study, only 28 (14%) had procured these from health facilities whilst the remaining 173 (86%) had procured their contraception from street vendors, non-governmental organisations and local pharmaceutical stores.

### Household income

The highest proportion of respondents who utilised the four maternal healthcare services in AMAC were in the lower income category. Unlike earlier reports (NBS *et al*. 2013:130–145; NPC & ICF Macro 2009:71–136), this study did not establish a pattern of service utilisation along household income categories. A lower proportion of respondents in the average income category compared with those in the higher income category utilised the four maternal healthcare services. In addition, only the differential in the use of modern contraceptive methods (*p* = 0.017) amongst women in different income categories was significant. The use of ANC (*p* = 0.294), skilled delivery care (*p* = 0.080) and PNC (*p* = 0.104) were not influenced significantly by income categories of the respondents.

### Description of inequality in maternal healthcare service utilisation

The median direct payment for maternal healthcare service utilisation amongst respondents was Naira 2150.00; and ranged from no payment to Naira 252 000.00. The concentration index (CI) for each of the four maternal healthcare services (Table 5), however, depict inequality that favours the rich (CI was positive) in service utilisation. The inequality was more pronounced in the use of modern contraceptive methods (CI = 0.06) compared to ANC (CI = 0.02), skilled delivery care (CI = 0.03) and PNC (CI = 0.03). These findings might be related to the fact that the median annual household income of Naira 699 996.00 amongst the respondents was higher than the annual national average income in Nigeria (Federal Government of Nigeria 2011:3).

**TABLE 5:** Inequality in maternal healthcare service utilisation against income group of respondents.

Utilisation of maternal health service	Concentration index (CI)
Used modern contraceptive methods.	0.059†
Received ANC from skilled medical providers.	0.016†
Had skilled attendants at birth.	0.030†
Receive PNC from medical personnel.	0.028†

†, concentration index is positive; ANC, antenatal care; PNC, postnatal care.

**FIGURE 2:**
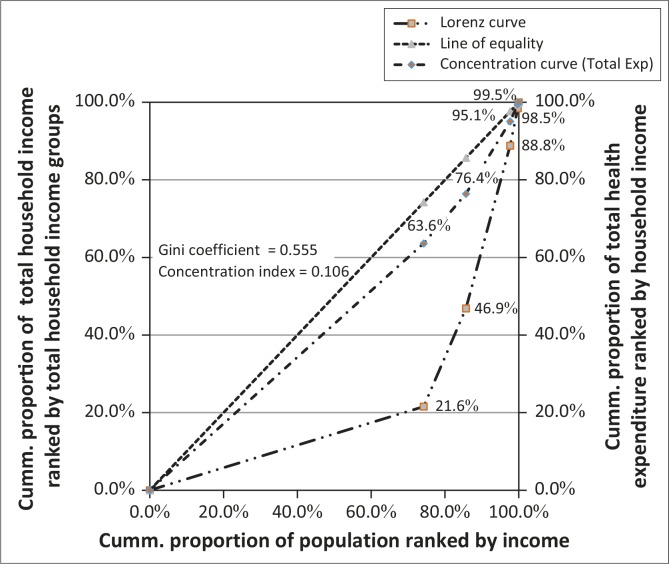
The Lorenz curve of income and the direct maternal healthcare expenditure concentration curve.

In Figure 2, the Lorenz curve (Gini-coefficient = 0.555) indicated that the distribution of total household income was not equitable amongst the respondents. The Kakwani index, calculated as the difference between CI of direct payment and Gini-coefficient (0.106–0.555) was -0.449. Since *K* < 1 and the direct payment concentration curve lay above the Lorenz curve, the analysis indicated that respondents in lower income categories spent more than their total income share on health and those in higher income categories spent less than their share. The payment system for maternal healthcare service utilisation in AMAC was therefore regressive and unfair on the poor.

### Socioeconomic predictor of maternal healthcare service utilisation

The independent effects (bivariate analysis) of the six SECs included in this study on maternal healthcare service utilisation varied when analysed, controlling for the effects of other independent variables in the logistic regression (Table 6).

**TABLE 6:** Predictors of utilisation of maternal healthcare services in Abuja Municipal Areas Council, Abuja, Nigeria (Logistic regression model).

Variable	Contraceptive services		Antenatal care service		Delivery care service		Postnatal care service	
	*p*-value	Odds Ratio – Exp β (95% CI)	*p*-value	Odds Ratio – Exp β (95% CI)	*p*-value	Odds Ratio – Exp β (95% CI)	*p*-value	Odds Ratio – Exp β (95% CI)
**Age**								
Younger than 20 years	0.256	1.00	0.000	1.00	0.009	1.00	0.004	1.00
20–34 years	0.157	0.32 (0.07 – 1.56)	0.000	0.023 (0.00 – 0.15)	0.003	0.1 (0.02 – 0.46)	0.003	0.1 (0.02 – 0.46)
35 years and older	0.297	0.42 (0.08 – 2.17)	0.000	0.03 (0.01 – 0.23)	0.015	0.13 (0.03 – 0.68)	0.026	0.16 (0.03 – 0.81)
**Education**								
None / Primary	0	1.00	0.021	1.00	0.130	1.00	0.058	1.00
Secondary	0.191	0.45 (0.14 – 1.48)	0.006	0.19 (0.06 – 0.61)	0.047	0.38 (0.15 – 0.99)	0.026	0.34 (0.13 – 0.89)
Post-secondary	0.001	0.14 (0.05 – 0.44)	0.017	0.26 (0.08 – 0.78)	0.063	0.42 (0.17 – 1.05)	0.019	0.33 (0.13 – 0.83)
**Location of residence**								
Rural	-	1.00	-	1.00	-	1.00	-	1.00
Urban	0.008	0.35 (0.16 – 0.76)	0.067	2.43 (0.94 – 6.30)	0.493	1.27 (0.64 – 2.53)	0.338	1.4 (0.70 – 2.79)
**Income group**								
Lower	0.313	1.00	0.251	1.00	0.196	1.00	0.224	1.00
Average	0.295	0.69 (0.35 – 1.38)	0.220	1.71 (0.73 – 4.02)	0.568	1.23 (0.61 – 2.47)	0.403	1.34 (0.67 – 2.68)
Higher	0.21	0.66 (0.35 – 1.26)	0.362	0.67 (0.28 – 1.60)	0.107	0.55 (0.27 – 1.14)	0.171	0.61 (0.30 – 1.24)
**Birth order**								
Once	**-**	1.00	**-**	1.00	**-**	1.00	**-**	1.00
More than once	0.849	0.96 (0.61 – 1.51)	0.000	0.1 (0.06 – 0.19)	0.000	0.41 (0.26 – 0.65)	0.000	0.43 (0.27 – 0.69)
**Insurance coverage**								
Not covered	**-**	1.00	-	1.00	-	1.00	-	1.00
Covered	0.574	1.16 (0.69 – 1.96)	0.272	0.66 (0.31 – 1.39)	0.007	0.43 (0.23 – 0.79)	0.016	0.48 (0.27 – 0.87)
**Constant**	0.000	34.03	0.000	54.77	0.001	19.26	0.001	20.17
Total number of cases		384		384		384		384
Model chi-square (*df* = 9)		65.85		106.96		52.94		50.72
Model significance (*p*-value)		0.000		0.000		0.000		0.000
-2 log likelihood		465.98		308.84		445.04		451.97

CI, confidence interval.

### Predisposing factors

Maternal age, birth order and maternal education were the predisposing factors included in this study. The influence of maternal age and birth order on the use of ANC, skilled delivery care and PNC were consistent in both the bivariate and multivariate analyses and similar to findings in Nigeria (Awusi *et al*. 2009:22) and Ethiopia (Mekonnen & Mekonnen 2003:375). Whilst the influence of birth order on the use of modern contraceptive methods was consistent, that for maternal age was contradictory. In AMAC, maternal age was established as a significant predisposing factor for the use of ANC (*p* = 0.000), skilled delivery care (*p* = 0.009) and PNC (*p* = 0.004). This study, however, indicated that older women were less likely than adolescent women (the reference group) to use the four maternal healthcare services. Exposure to better information on healthcare and limited pregnancy-related experience (a motivating factor) amongst younger women could contribute to the observed pattern. A significant predisposing effect of birth order on the use of ANC (*p* = 0.000), skilled delivery care (*p* = 0.000) and PNC (*p* = 0.000) was established. There were, however, concerns that respondents who had experienced more than one birth had a lower likelihood of utilising maternal healthcare services. This observation might be related to the (perceived) poor quality of care received, which could be a disincentive for continuing service utilisation. The predisposing effects of maternal age (*p* = 0.256) and birth order (*p* = 0.849) on the use of modern contraceptive methods were not significant.

The influence of maternal education was consistent for the use of modern contraceptive methods in the bivariate and multivariate analyses, but contradictory for the use of ANC, skilled delivery care and PNC services. Maternal education was, however, established as a significant predisposing factor for the use of modern contraceptive methods (*p* = 0.000) and ANC (*p* = 0.021) in AMAC. The predisposing effect of maternal education on the use of skilled delivery care (*p* = 0.130) and PNC (*p* = 0.058) was not significant. Unlike earlier studies (Adanu 2010:155; Butawa *et al*. 2010:75), women with a higher level of education were less predisposed than those with no or a primary level of education (reference group) to utilise the four maternal healthcare services. Although not of statistical significance (*p*-value > 0.05), a similar (negative) predisposing effect of maternal education on the use of family planning services (OR = 0.001) was reported in Kenya (Okech *et al*. 2011:34).

### Enabling factors

This study included location of residence, health insurance coverage and household income as enabling factors for maternal healthcare service utilisation. The influence of location of residence was consistent for the use of the four maternal healthcare services in the bivariate and multivariate analyses. Location of residence was established as a significant enabling factor for the utilisation of modern contraceptive methods (*p* = 0.008) in AMAC. Although the enabling effect of location of residence for the use of ANC (*p* = 0.067), skilled delivery care (*p* = 0.493) and PNC (*p* = 0.338) was not statistically significant, the findings of this study suggest that respondents in urban areas had a higher likelihood of utilising the three maternal healthcare services (ANC, skilled delivery care and PNC). Women in urban areas of AMAC, however, had a lower likelihood of using modern contraceptive methods when compared to women in rural areas (reference group). A mixed effect of location of residence on the utilisation of the different maternal healthcare services were reported across several studies (Mekonnen & Mekonnen 2003:376–378; Say & Raine 2007:814; Zere *et al*. 2010:5), with an overall pattern in favour of urban women for delivery care and a somewhat mixed pattern for ANC service.

The influence of health insurance coverage was consistent for the use of modern contraceptive methods, skilled delivery care and PNC service in the bivariate and multivariate analyses, but contradictory for the use of ANC. In AMAC, health insurance coverage was established as a significant enabling factor for the use of skilled delivery care (*p* = 0.007) and PNC (*p* = 0.016). However, the likelihood of utilising health facility-based services (ANC, delivery care and PNC) was lower amongst respondents covered by health insurance when compared with those who were not (reference group). In contrast, respondents covered by health insurance had a higher likelihood of using modern contraceptive methods procured largely outside the health facilities. The enabling effect of health insurance coverage on the use of modern contraceptive methods (*p* = 0.574) and ANC (*p* = 0.16) was not significant. In addition to the fact that only 91 (24%) respondents were covered by health insurance, the limited benefit coverage of health insurance schemes in low- and middle-income countries (Ibiwoye & Adeleke 2000:220; Meng *et al*. 2011:94) might explain the observed effect.

The influence of household income was consistent for the use of ANC, skilled delivery care and PNC in the bivariate and multivariate analyses, but contradictory for the use of modern contraceptive methods. Household income was, however, not established as a significant enabling factor for the use of modern contraceptive methods (*p* = 0.313), ANC (*p* = 0.251), skilled delivery care (*p* = 0.196) and PNC (*p* = 0.224) in AMAC. These findings were consistent with the argument in Wilkinson (1997:593), stating that absolute income was unrelated to health. This study suggest that women in the higher income category were less likely to utilise the four maternal healthcare services when compared with those in the lower income category (reference group). However, respondents in the average income category had a higher likelihood of using ANC, skilled delivery care and PNC services when compared to the reference group.

## Limitations of the study

Although this study was limited to pregnant women attending ANC in the five district hospitals in AMAC, the fact that AMAC constituted 55% of the total population of Abuja (NPC 2010:36), as well as the large sample size calculated for the study, strengthened the application of the findings in Abuja.

The descriptive nature of the research design limits the establishment of causal relationships between SECs and the utilisation of maternal healthcare services. However, the multivariate analysis in this research indicated the effect of different respondent SECs as being predisposing and enabling factors (predictors) of the utilisation of maternal healthcare services.

## Recommendations

Policy measures and programme actions targeted at the multiple SEC of women in their natural co-existing state, as opposed to independent actions, are recommended to optimise maternal health benefits in AMAC. Within the comprehensive measure, there is a need to broaden the coverage of maternal healthcare services in the National Health Insurance Scheme (NHIS). Such actions should seek to eliminate the regressive payment system and contribute to resolving the inequality in the utilisation of maternal healthcare services. In addition, social mobilisation programmes targeting adolescents and women who have experienced more than one birth should be prioritised so as to address demand-side concerns in relation to access and utilisation of services. This should be complemented with quality-improvement initiatives to address potential service quality gaps. A review of the implementation approach for contraceptive services in AMAC is recommended in order to take advantage of the predictive effects of maternal education and location of residence to improve uptake.

Policy research on healthcare financing in AMAC is required to provide additional insight into the strengths and weaknesses of the different financing options for maternal healthcare services to guide necessary reforms. A clinical review of the quality of the four maternal healthcare services in AMAC is recommended to identify gaps in the service performance against national and international standards. The review would serve to both reinforce good practices and address quality gaps.

## Conclusion

This study established factors contributing to the exclusion of women from maternal health benefit in AMAC. In establishing the factors, the conceptual framework was useful in defining the four maternal healthcare services required to achieve maternal health benefit. The findings of this study are, however, some of the few that challenged the expectation that women who were older, more educated, with higher birth order, or resident in urban areas, have a higher likelihood of utilising maternal healthcare services. As such, this research opens up new policy and programme considerations as well as further studies required to optimise utilisation of maternal healthcare services.
